# Generation and repair of thymic epithelial cells

**DOI:** 10.1084/jem.20230894

**Published:** 2024-07-09

**Authors:** Graham Anderson, Emilie J. Cosway, Kieran D. James, Izumi Ohigashi, Yousuke Takahama

**Affiliations:** 1 Institute for Immunology and Immunotherapy, University of Birmingham, Birmingham, UK; 2 Division of Experimental Immunology, Institute of Advanced Medical Sciences, University of Tokushima, Tokushima, Japan; 3 Thymus Biology Section, Experimental Immunology Branch, National Cancer Institute, National Institutes of Health, Bethesda, MD, USA

## Abstract

In the vertebrate immune system, thymus stromal microenvironments support the generation of αβT cells from immature thymocytes. Thymic epithelial cells are of particular importance, and the generation of cortical and medullary epithelial lineages from progenitor stages controls the initiation and maintenance of thymus function. Here, we discuss the developmental pathways that regulate thymic epithelial cell diversity during both the embryonic and postnatal periods. We also examine how thymus microenvironments respond to injury, with particular focus on mechanisms that ensure regeneration of thymic epithelial cells for the restoration of thymus function.

## Teaser

Thymic epithelial cells (TECs) are essential for T-cell development, and their injury limits thymus function. We explain how TEC development creates multifunctional cortical and medullary microenvironments and describe regenerative pathways where TEC targeting creates therapeutic opportunities to restore T-cell immunity.

## Introduction

The thymus is an epithelial–mesenchymal organ unique in its ability to support self-tolerant, MHC-restricted αβT cell development. During embryogenesis in the mouse, thymic epithelial cells (TECs) arise from endodermal cells within the third pharyngeal pouch (Gordon et al., 2004; Rodewald, 2008). Early in thymus organogenesis, expression of the master transcription factor *Foxn1* is initiated, which is essential for continued development of the thymus rudiment (Blackburn et al., 1996; Nehls et al., 1994). Epithelial cell expression of *Foxn1* supports the induction of a TEC developmental program involving proliferation and differentiation (Nehls et al., 1996; Nowell et al., 2011; Su et al., 2003), leading to formation of cortex and medulla areas containing cortical TECs (cTECs) and medullary TECs (mTECs) (Bosticardo et al., 2021; Han and Zúñiga-Pflücker, 2021). Importantly, *Foxn1* expression controls a panel of genes that represent key regulators of thymopoiesis, including *Ccl25* and *Cxcl12* (for lymphoid progenitor recruitment), *Dll4* (for T-cell specification and development), and *Cd8*3 and β5t-encoding *Psmb11* (for thymic selection) (Bleul and Boehm, 2000; Ripen et al., 2011; Uddin et al., 2017; Žuklys et al., 2016). The importance of TECs for T-cell development is clear from studies in both mice and humans, where detrimental mutations in key genes (e.g., *Foxn1*, *Tbx1*, *Pax1*, and *Foxi3*) disrupt thymus organogenesis and cause either partial or complete loss of thymus tissue and T-cell immunodeficiency (Bosticardo and Notarangelo, 2023; Kreins et al., 2021; Vaidya et al., 2016).

Despite sharing a common origin of third pharyngeal pouch endoderm during embryonic development (Gordon et al., 2004), TECs in the adult thymus are highly heterogeneous. Moreover, several studies point toward cellular renewal within both cTECs and mTECs (Dumont-Lagacé et al., 2017; Gray et al., 2006; Michel et al., 2018), suggesting that TECs undergo turnover in the steady-state adult thymus. Collectively, these observations indicate TEC developmental pathways where stem and/or progenitor populations control the formation, maintenance, and turnover of cTECs and mTECs. It is important to note that various TEC progenitors have been reported in both the embryo and postnatal thymus (Farley et al., 2023; Lucas et al., 2023; Ragazzini et al., 2023; Rodewald et al., 2001), including bipotent progenitors that give rise to both cTECs and mTECs (Bleul et al., 2006; Nusser et al., 2022; Rossi et al., 2006). However, it is unknown whether developmental pathways leading to cortex and medulla formation in the embryo are the same as those that maintain postnatal thymus tissue. Indeed, recent studies (Nusser et al., 2022) indicate a bias of bipotent progenitors in the embryo to cTECs and a bias of postnatal bipotent progenitors to mTECs, suggesting embryonic and adult TEC developmental pathways are different.

Regarding TEC maintenance during postnatal stages, an interesting feature of thymus tissue is its capacity for repair following injury. Thymus injury may be a consequence of physiological stimuli including infection, stress, or malnutrition, or clinical interventions such as chemotherapy or radiotherapy used for disease treatment (Dooley and Liston, 2012; Velardi et al., 2021). In these scenarios, recovery requires re-establishment of functionally competent TECs to restore self-tolerant T-cell immunity (Cowan et al., 2020). Whether the processes controlling TEC regeneration parallel those controlling steady state TEC development is unclear. However, it is interesting that just as multiple hemopoietic cells (e.g., ILC3, invariant natural killer T cells [iNKT], conventional αβT cells and γδT cells) control steady-state TECs (Hikosaka et al., 2008; Irla et al., 2008; Roberts et al., 2012; Rossi et al., 2007a; White et al., 2014), crosstalk with hemopoietic cells (e.g., ILC3, ILC2, eosinophils) is important for TEC regeneration (Cosway et al., 2022; Dudakov et al., 2012).

In this review, we examine progress in understanding how TEC development is initiated during embryogenesis and maintained into adulthood. We pay particular attention to how heterogeneity within cTECs and mTECs and progenitor/stem compartments relates to developmental pathways of TEC development. Finally, we describe mechanisms controlling TEC re-establishment following injury, which may eventually point toward a better understanding of the processes controlling TECs in health and disease.

## TEC development and diversification

### TEC progenitors

#### Embryonic TEC development and Foxn1

The thymus originates from the endoderm of the pharyngeal pouch during embryogenesis (Blackburn and Manley, 2004). In mice and humans, the thymus derives from the third pharyngeal pouch (Boyd, 1950). In clawed frogs, the thymus derives from the second pharyngeal pouch, and in chicken, from the third and fourth pouches (Hilfer and Brown, 1984; Lee et al., 2013). Emergence of TECs is initiated on embryonic day (E)11 in mice and mid-week 6 in humans and is detectable by expression of the landmark transcription factor *Foxn1* (Gordon et al., 2001), the gene responsible for the athymic phenotype in *nude* mice (Nehls et al., 1994). By E11, TECs form multilayered stratified structures like other epithelia such as the skin epidermis (Gordon et al., 2004; Hamazaki et al., 2007). This initial formation of the thymus anlage does not require *Foxn1* (Nehls et al., 1996). Subsequently, TECs form a three-dimensional meshwork structure that provides a functional microenvironment to support T-cell development and selection. The development of functional thymus is dependent on *Foxn1* in TECs (Nehls et al., 1996). The promoter activity for *Foxn1* transcription and the frequency of FOXN1^+^ cells in TECs are high during embryogenesis and decline after birth (Itoi et al., 2007; Rode et al., 2015; Rota et al., 2021). However, the importance of *Foxn1* is not limited to embryonic thymus development. Forced reduction of *Foxn1* expression in the postnatal thymus resulted in a decrease in TEC proliferation and disorganization of both cortical and medullary compartments, indicating the persistent contribution of *Foxn1* in maintaining the postnatal thymic microenvironment (Chen et al., 2009). Further analysis of a reverting mutant allele of *Foxn1* indicated that *Foxn1*-deficient TECs retain developmental capability in the postnatal period and that the reverted *Foxn1* expression in TECs is capable of promoting the formation of functionally potent thymus with cortical and medullary architecture (Bleul et al., 2006).

#### Bipotent TEC progenitors

**Figure 1.**
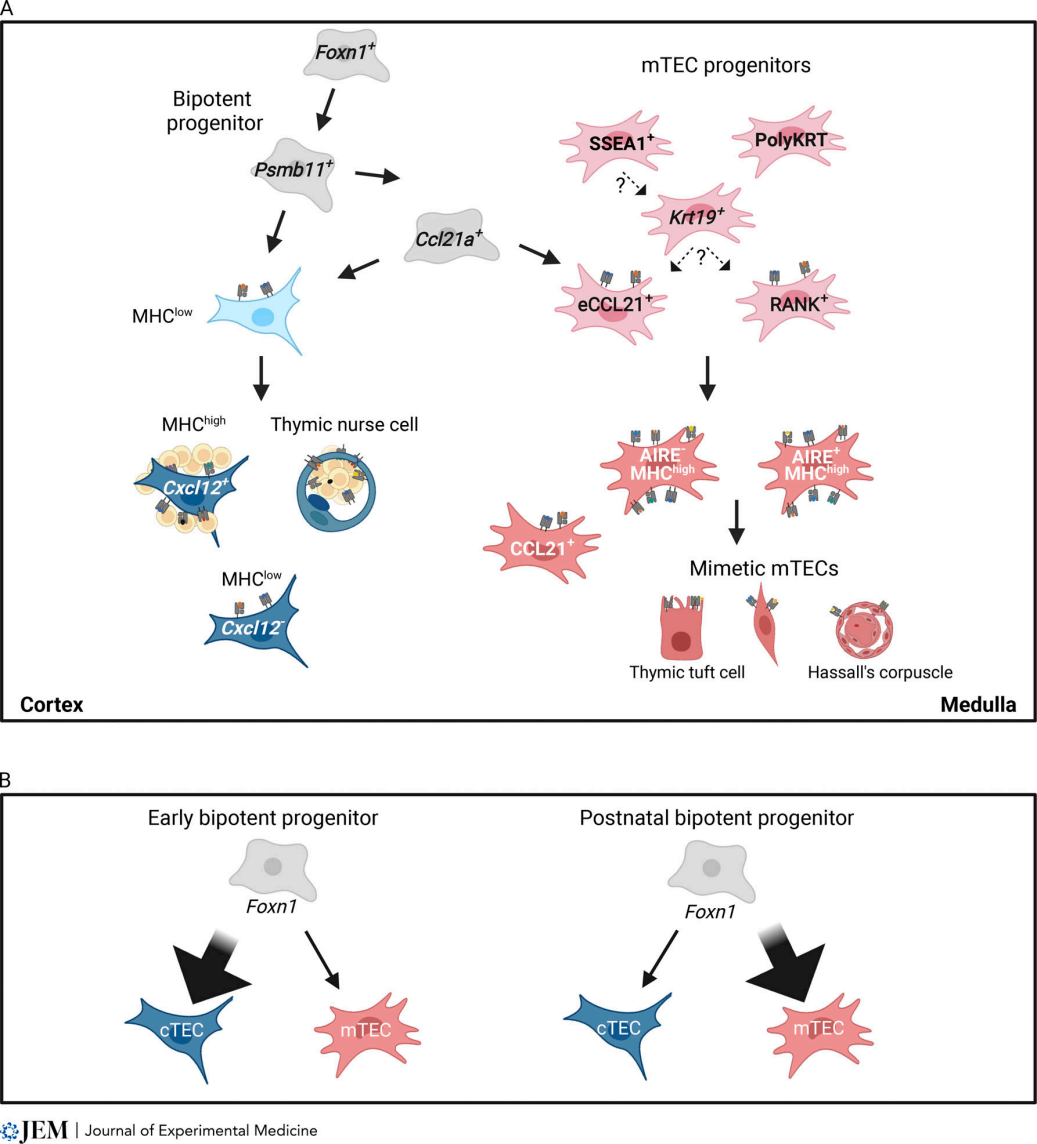
**Developmental pathways for heterogeneous cTECs and mTECs. (A)** cTECs and mTECs are derived from bipotent progenitors expressing cTEC-trait molecules such as *Psmb11* (β5t). cTEC development is mediated through the MHC^low^ stage. cTECs in postnatal thymus include MHC^high^ cTECs and TNCs, both of which are highly associated with cortical thymocytes, as well as late-appearing MHC^low^ cTECs. Expression of *Cxcl12* also subdivides cTEC in the adult thymus. Two-thirds of cTECs and the vast majority of mTECs are derived from cells that transcribe *Ccl21a*. CCL21-protein-expressing thymocyte-attracting functional mTECs in embryonic thymus (eCCL21^+^) retain progenitor potential. mTEC-restricted progenitors also include cells that express Krt19, SSEA1, RANK, and polykeratin (PolyKRT). Mature functional mTECs include thymocyte-attracting CCL21^+^ mTECs and self-antigen-displaying mTECs, including Aire^+^ and Aire^−^ MHC^high^ mTECs and a variety of mimetic mTECs. **(B)** Early progenitors preferentially give rise to cTECs, whereas postnatal progenitors are biased to become mTECs. Postnatal progenitors include cells that transcribe *Ccl21a*.

Lineage tracing and single-cell reconstitution studies showed embryonic TECs contain bipotent progenitor cells capable of differentiating into both cTECs and mTECs (Bleul et al., 2006; Rossi et al., 2006). Subsequent studies demonstrated cTECs and mTECs are derived from bipotent progenitors expressing cTEC-associated molecules, including β5t, CD205, and high levels of IL-7 (Baik et al., 2013; Ohigashi et al., 2013; Ribeiro et al., 2013). These findings led to the concept that TECs undergo a serial progression from uncommitted TEC progenitors to transitional TEC progenitors that express cTEC-associated molecules before the developmental diversification to either cTECs or mTECs (Alves et al., 2014; Takahama et al., 2017). Tracing the fate of β5t-expressing TECs revealed the majority of mTECs in the adult thymus are maintained and regenerated by the supply of embryonic and perinatal, rather than adult, β5t^+^ progenitors (Ohigashi et al., 2015; Mayer et al., 2016) (Fig. 1 A). Nonetheless, bipotent TEC progenitors capable of differentiating into both cTECs and mTECs have been detected in the adult thymus, either within α6-integrin^high^Sca-1^high^Ly51^low^MHCII^low^ TECs or Plet1^+^Ly51^+^MHCII^high^ TECs (Ulyanchenko et al., 2016; Wong et al., 2014). These different phenotypes may reflect heterogeneity in adult bipotent TEC progenitors. Recent studies have identified TEC stem cells in the postnatal human thymus capable of long-term expansion and multilineage differentiation and are characterized by expression of multiple keratin species (Campinoti et al., 2020; Ragazzini et al., 2023), resembling previously identified mouse TEC progenitors expressing both cTEC-associated keratin 8 and mTEC-associated keratin 5 (Klug et al., 1998).

#### Developmental progression of TEC progenitors

The cortex occupies most of the thymus during embryogenesis and the medulla expands during subsequent postnatal stages. The unequal development of cTECs and mTECs from common bipotent progenitors is in part due to the dependence of mTEC development on receptor activator of nuclear factor κΒ ligand (RANKL), CD40L, and lymphotoxin-mediated “thymic crosstalk” signals derived from late-appearing mature TCR^high^ thymocytes (Boehm et al., 2003; Akiyama et al., 2008; Hikosaka et al., 2008; Roberts et al., 2012; White et al., 2014). Recent studies demonstrated an additional mechanism of a shift in the developmental preference of TEC progenitors during ontogeny. Farley and colleagues examined the fate of Plet1^+^ TEC progenitors isolated from E11.5 or E12.5 mice that constitutively express GFP. Here, embryonic GFP^+^ TECs transplanted into embryonic thymic lobes of WT mice were preferentially detected in the cortex rather than medulla, suggesting embryonic TEC progenitors preferentially differentiate into cTECs rather than mTECs (Farley et al., 2023). Furthermore, using a combination of single-cell RNA-sequencing analysis of embryonic and postnatal TECs together with in vivo fate mapping of endogenously barcoded progenitors, Nusser et al. demonstrated that embryonic TEC progenitors preferentially gave rise to cTECs, whereas postnatal TEC progenitors were biased toward mTECs, indicating differences in developmental potential between early and postnatal TEC progenitors (Nusser et al., 2022) (Fig. 1 B).

It is interesting to note that in the embryonic thymus, TECs expressing cTEC-associated β5t emerge in the outer region of the thymus primordium, whereas TECs expressing mTEC-associated claudins-3 and -4 mostly localize in the inner region of the thymus primordium (Hamazaki et al., 2007; Ripen et al., 2011). The anatomically segregated distribution of cTEC- and mTEC-biased TECs may reflect the differential influence of local signals, for example, those derived from neighboring mesenchymal cells, on the specification to develop into cTECs and mTECs.

### Development and diversity of cTECs

#### Embryonic development of cTECs

cTECs primarily contribute to early T-cell development and positive selection of developing thymocytes. cTECs express various functional molecules for inducing early T-cell development, such as DLL4 and IL7 (Koch et al., 2008; Hozumi et al., 2008; Ribeiro et al., 2013), and for promoting positive selection of thymocytes, such as the β5t-containing thymoproteasome, cathepsin L, and Prss16, and MHC class I and class II molecules (Honey et al., 2002; Murata et al., 2007; Gommeaux et al., 2009). cTECs appear as early as E12, detected by expression of the cTEC-associated molecules β5t and CD205 (Shakib et al., 2009; Ripen et al., 2011). During embryonic development, the thymus primarily supports early T-cell development and positive selection, which coincides with the predominance of cTECs over mTECs in the embryonic thymus. In contrast, mTECs are important for supporting late-stage T-cell development including negative selection to establish self-tolerance in T cells. Accordingly, mTECs remain minor in TEC cellularity during embryogenesis and subsequently increase during perinatal and postnatal development of the thymus.

The developmental maturation of cTECs during embryogenesis can be monitored by the expression of CD205 and CD40. Early cTEC development is detectable as CD205^+^CD40^−^ and then CD205^+^CD40^+^ cells expressing high levels of MHC class II molecules (Shakib et al., 2009). This development of cTECs requires the concomitant development of thymocytes, as human CD3ε transgenic tgε26 mice in which thymocyte development is arrested at an early CD4^−^CD8^−^CD44^+^CD25^−^ DN1 stage (Wang et al., 1994; Holländer et al., 1995), showed the developmental arrest of cTECs at the CD205^+^CD40^−^ stage (Shakib et al., 2009). cTEC maturation during embryogenesis is also accompanied by elevated expression of CCRL1, an atypical chemokine receptor capable of scavenging CCL19, CCL21, and CCL25 (Ribeiro et al., 2014). This CCRL1 elevation in cTECs is diminished in Rag2/IL2Rγ double-deficient mice, in which virtually no CD45^+^ thymocytes are detectable (Ribeiro et al., 2014). Thus, bilateral signals between cTECs and thymocytes promote their symbiotic development.

#### Postnatal development of cTEC heterogeneity

Studies have revealed heterogeneity within cTECs in the postnatal thymus. Perhaps the most classical definition is detection of thymic nurse cells (TNCs) within the thymic cortex in situ (Kyewski and Kaplan 1982; Nakagawa et al., 2012; Venables et al., 2019) (Fig. 1 A). TNCs represent a large fraction of postnatal cTECs, tightly interacting with many CD4^+^CD8^+^ (DP) thymocytes to support their survival and secondary TCRα rearrangement (Nakagawa et al., 2012). The morphology of these cTECs, which extend multiple cell projections and wrap around multiple thymocytes, makes it difficult to isolate viable cTECs by mechanical and enzymatic digestion, causing a drastic underestimation of cTECs isolated in cell suspension studies, typically <1% of total cTEC numbers (Sakata et al., 2018; Hirakawa et al., 2018; Venebles et al., 2019).

Using a reporter mouse in which *Cxcl12* expression is monitored by the fluorescent protein dsRed, a recent study disclosed that ∼40% of cTECs in adult mice are negative for *Cxcl12*^dsRed^ expression (White et al., 2022). This heterogeneity in postnatal cTECs sharply contrasts the homogeneous expression of *Cxcl12*^dsRed^ in the vast majority of newborn cTECs. Postnatal *Cxcl12*^dsRed−^ cTECs lack expression of *Foxn1* and *Foxn1*-dependent genes, including *Cxcl12*, *Dll4*, *Ccl25*, *Psmb11*, and *Prss16*. However, *Cxcl12*^dsRed−^ cTECs are derived from *Foxn1*-expressing TECs (White et al., 2022). Likewise, the majority of embryonic TECs express *Foxn1*, whereas TECs lacking *Foxn1* emerge postnatally and are derived from *Foxn1*-expressing cells (Rode et al., 2015; O’Neill et al., 2016). The cTEC heterogeneity in the adult thymus emerges in a thymocyte-dependent manner, namely, the development of *Cxcl12*^dsRed−^ cTECs is impaired in *Rag2*-deficient mice, in which thymocyte development is arrested at the CD4^−^CD8^−^CD44^−^CD25^+^ DN3 stage, suggesting that developing thymocytes promote the development of *Cxcl12*^dsRed−^ cTECs (White et al., 2022). Interestingly, in comparison with *Cxcl12*^dsRed+^ cTECs, *Cxcl12*^dsRed−^ cTECs are significantly less capable of interacting with thymocytes, suggesting that the promotion of thymocyte development by *Cxcl12*^dsRed+^ cTECs subsequently drives the postnatal development of *Cxcl12*^dsRed−^ cTECs (Fig. 1 A). Another study also showed that DLL4, which is highly expressed in embryonic cTECs, declines postnatally in a manner dependent on developing thymocytes (Fiorini et al., 2008). Given that DLL4 is controlled by *Foxn1*, this loss of DLL4 expression by cTEC in the adult thymus correlates well with the progressive appearance of *Foxn1*^*−*^ cTEC that also lack expression of Foxn1 target genes including *Cxcl12* (White et al., 2022).

Single-cell transcriptomic analyses have also verified cTEC heterogeneity. Baran-Gale and colleagues identified two distinct cTEC subpopulations in postnatal thymus; one population termed perinatal cTECs was abundant during the perinatal period and decreased thereafter, whereas another population termed mature cTECs was increased in the adult thymus (Baran-Gale et al., 2020). Notably, the frequency of perinatal cTECs remained high in adult *Rag2*-deficient mice, while administration of anti-CD3 antibody to *Rag2*-deficient mice to induce DP thymocyte development resulted in a decrease in the perinatal cTEC population (Klein et al., 2023). These findings further support the idea that cTEC heterogeneity arises as a result of the perinatal cTEC-mediated generation of DP thymocytes. It will be interesting to identify thymocyte-derived signals that drive the development of postnatal cTEC subpopulations. It is also important to understand the function of those postnatal cTEC subpopulations.

### Diversity and development of mTECs

#### Diversity of mTECs

mTECs primarily contribute to the installment of self-tolerance in T cells. The thymic process for T-cell self-tolerance, also known as central tolerance, is supported not only by mTECs but also by multiple antigen-presenting cells including dendritic cells (DCs) and B cells (Hubert et al., 2011; Marx et al., 2021; Inglesfield et al., 2019). However, the role of mTECs in central tolerance is evident by the onset of autoimmune disease in mice deficient in mTECs (Cowan et al., 2013; Riemann et al., 2017). For example, *Foxn1*-mediated conditional deletion of the transcription factor *Relb* in TECs causes a specific loss of mTECs in the mouse and results in the development of spontaneous autoimmune lesions in multiple tissues along with the production of autoantibodies (Riemann et al., 2017). Moreover, grafting of *Relb*-deficient TECs into athymic nude mice resulted in autoimmunity associated with a significant reduction in the development of Foxp3^+^ regulatory T cells (Cowan et al., 2013). Thus, mTECs are essential to prevent autoimmunity and to establish central tolerance.

However, mTECs are highly heterogeneous in their functions and morphology. The function of mTECs can be categorized into two aspects in terms of the regulation of T-cell development and selection. First, mTECs have the machinery to transcribe a variety of genomic components including tissue-restricted self-antigen molecules, so that developing thymocytes can encounter and establish self-tolerance to virtually all self-antigens encoded in the genome. Second, mTECs produce chemokines and signaling molecules to attract and deploy hematopoietic cells including developing thymocytes into and out of the thymic medulla. Both functions are essential for the establishment of self-tolerance in newly generated T cells. The cooperation of functionally diverse mTECs contributes to self-tolerance by promoting the elimination of self-reactive thymocytes and by promoting the development of regulatory T cells (Ushio et al., 2024).

Interestingly, these functions of mTECs are mediated largely by distinct mTEC subpopulations. Self-antigen-displaying mTECs are heterogeneous and include Aire-expressing MHC class II^high^ mTECs (mTEC^high^) and mimetic mTECs expressing low levels of MHC class II molecules (mTEC^low^). Thymocyte-attracting CCL21-expressing mTECs comprise another functional mTEC^low^ subpopulation (Lkhagvasuren et al., 2013; Ohigashi et al., 2024). Eosinophil-attracting CCL11-producing type 2 cytokine receptor-expressing mTECs, which regulate the emigration of mature thymocytes and the recruitment of eosinophils during thymus regeneration, are also included predominantly in mTEC^low^ (White et al., 2017; Cosway et al., 2022). Chemokine XCL1-producing mTECs, which contribute to the accumulation of DCs in the thymic medulla for T-cell self-tolerance, are generated in an Aire-dependent manner (Lei et al., 2011).

#### Development and developmental potential of Aire-expressing mTECs

The promiscuous expression of tissue-restricted antigens in the thymic medulla, which was discovered from the thymic expression of pancreas-specific insulin and acute-phase liver-specific C-reactive protein (Jolicoeur et al., 1994; Smith et al., 1997; Klein et al., 1998; Klein and Kyewski, 2000), is mediated at least in part by the nuclear protein AIRE expressed in the mTEC^high^ subpopulation (Derbinski et al., 2001; Anderson et al., 2002). *Aire*-expressing mTEC^high^ are mosaics in terms of promiscuous gene expression. The estimated frequency of promiscuously expressed self-antigen genes in *Aire*-expressing mTEC^high^ is between 2 and 15% depending on the genes (Derbinski et al., 2008; Sansom et al., 2014). Reaggregate thymus organ culture experiments showed that mTEC^high^, including *Aire*-expressing mTECs, are derived from embryonic and postnatal mTEC^low^, indicating that mTEC^low^ include cells with a developmental potential to give rise to mTEC^high^ (Gray et al., 2006, 2007; Rossi et al., 2007a; Gäbler et al., 2007). *Aire*-independent self-antigen expression is also detected primarily in mTEC^high^ (Derbinski et al., 2005; Sansom et al., 2014) and is in part regulated by the transcription factor *Fezf2* (Takaba et al., 2015).

The lack of proliferative potential in *Aire*-expressing mTECs in the postnatal thymus and the apoptosis of an mTEC cell line by AIRE overexpression suggested *Aire*-expressing mTECs represent terminally differentiated mTECs (Gray et al., 2007). However, the postnatal increase in mTEC^low^ suggested that mTEC^low^ are not solely the progenitors of mTEC^high^ but include an additional population that accumulates postnatally (Gray et al., 2006). By lineage tracing of *Aire*-expressing cells, it was revealed that *Aire*-expressing mTECs are capable of further differentiation into *Aire*-negative mTEC^low^ (Nishikawa et al., 2010; Metzger et al., 2013), which includes recently described mimetic mTECs.

#### Heterogeneity and development of mimetic mTECs

Morphological diversity in mTECs was noted as early as the 19th century by the discovery of Hassall’s corpuscles with concentric whorls of stratified epithelial cells (Hassall, 1849). Findings of ciliated columnar epithelial cells and neurosecretory epithelial cells in the medulla documented further heterogeneity in mTEC morphology (Farr and Rudensky, 1998). Interestingly, these highly diverse and terminally differentiated epithelial cells localized in the medulla are at least in part detected in post-*Aire* mTECs (White et al., 2010; Metzger et al., 2013). More recent studies reported an mTEC subpopulation resembling intestinal tuft cells in morphology and molecular expression profiles, including expression of type 2 taste receptors and the type 2 cytokine IL-25 (Miller et al., 2018; Bornstein et al., 2018). Thymic tuft cells are partially derived from *Aire*-expressing mTECs, even though *Aire* is not necessary for thymic tuft cell development and not all thymic tuft cells derive from *Aire*-expressing mTECs (Miller et al., 2018). Like intestinal tuft cells, development of thymic tuft cells is dependent on the transcription factor *Pou2f3* (Miller et al., 2018; Bornstein et al., 2018). Recent studies further noted the role of Fezf2 in the development of thymic tuft cells (Lammers et al., 2024; Ushio et al., 2024).

Michelson and colleagues reconfirmed the diversity in the post-*Aire* mTEC^low^ population by chromatin accessibility assay and RNA-sequencing analysis of individual mTECs (Michelson et al., 2022). Each post-*Aire* cluster is enriched with the binding motif of transcription factors known to be essential for extrathymic tissues, reflecting the chromatin accessibility of genes specific to each extrathymic tissue driven by that transcription factor. These post-*Aire* mTECs mimic extrathymic cells and are suggested to be involved in T-cell tolerance to mimetic cell antigens, thereby termed mimetic mTECs (Michelson et al., 2022) (Fig. 1 A).

Mimetic mTECs may extend their functions beyond self-tolerance establishment in conventional T cells. Thymic tuft cells exhibit a role in regulating the development and function of invariant NKT2 cells in the thymus (Miller et al., 2018; Lucas et al., 2020). Another study reported that endocrine mimetic mTECs control the cellularity of the thymus in a ghrelin-dependent manner, and microfold mimetic mTECs regulate the generation of IgA^+^ plasma cells in the thymus (Givony et al., 2023).

#### Development and developmental potential of CCL21-expressing mTECs

The chemokine CCL21 produced by mTECs is critical for the establishment of T-cell tolerance through the chemoattraction of positively selected thymocytes from the cortex to the medulla (Kurobe et al., 2006; Kozai et al., 2017). CCL21 protein produced in the thymic medulla is also captured by mesenchymal stroma and contributes to neonatal T-cell emigration (James et al., 2021). Positive selection-inducing TCR signals in cortical thymocytes elevate the expression of CCR7, a receptor for CCL21, and positively selected cortical thymocytes are attracted to the medullary region through CCL21-CCR7–mediated chemotactic signals (Ueno et al., 2004). Two molecular species, CCL21Ser and CCL21Leu, with one amino acid difference are encoded in the mouse genome (Nakano and Gunn, 2001; Chen et al., 2002), and CCL21Ser encoded by *Ccl21a* locus is predominantly expressed in the thymic medulla (Kozai et al., 2017). In mice lacking either *Ccr7* or *Ccl21a*, positively selected mature thymocytes fail to accumulate in the medulla, and T cells fail to establish self-tolerance (Kurobe et al., 2006; Kozai et al., 2017). The additional CCR7-ligand CCL19 has no appreciable role in the cortex-to-medulla migration of developing thymocytes (Link et al., 2007; Kozai et al., 2017). Thus, CCL21-expressing thymocyte-attracting mTECs represent a functional mTEC subset essential for the establishment of self-tolerance in T cells.

CCL21^+^ mTECs are included in the mTEC^low^ subpopulation and distinct from AIRE^+^ mTECs (Lkhagvasuren et al., 2013; Kozai et al., 2017). Fate-mapping analysis indicated that *Ccl21a*^+^ mTECs have a developmental potential to generate most mTECs including AIRE^+^ mTECs and thymic tuft cells, and reaggregate thymus organ culture experiments confirmed that embryonic CCL21^+^ mTECs, which are functionally potent to accumulate medullary thymocytes in the embryonic thymus, can give rise to AIRE^+^ mTECs (Ohigashi et al., 2024). Thus, thymocyte-attracting CCL21^+^ mTECs in embryonic thymus include progenitor activity to become self-antigen-displaying mTECs, including AIRE^+^ mTECs. These findings also indicate the functional conversion of thymocyte-attracting mTECs into self-antigen-displaying mTECs contributes to the development of heterogenous mTEC subpopulations.

Fate-mapping analysis further showed approximately two-thirds (66%) of cTECs are derived from *Ccl21a*^+^ cells, suggesting *Ccl21a*-transcribing mTECs, which are detectable only in the thymic medulla, include the developmental potential equivalent to bipotent TEC progenitors. Indeed, cTECs derived from *Ccl21a*^+^ cells are enriched in the perimedullary region of the thymic cortex (Ohigashi et al., 2024). Interestingly, postnatally appearing mTEC-biased progenitors included cells transcribing *Ccl21a* (Nusser et al., 2022). These results suggest the similarity and potential overlap between the *Ccl21a*^+^ fraction of cTEC progenitors and postnatal mTEC-biased bipotent progenitors.

In contrast to embryonic CCL21^+^ mTECs, CCL21^+^ mTECs isolated from postnatal thymus failed to show the developmental potential to give rise to AIRE^+^ mTECs, and the gene expression profiles were markedly different between embryonic and postnatal CCL21^+^ mTECs (Ohigashi et al., 2024). Postnatal CCL21^+^ mTECs may include mTEC-biased bipotent progenitors, but the majority of postnatal CCL21^+^ mTECs represent terminally differentiated mTEC^low^ that lack progenitor potential (Fig. 1 A).

#### Heterogeneous mTEC progenitors

Heterogeneous subpopulations of functional mTECs originate from bipotent TEC progenitors, which are heterogeneous themselves in developmental stages, i.e., the stages before and after the acquisition of cTEC traits, and in developmental progression, i.e., embryonic cTEC-biased and postnatal mTEC-biased progenitors. cTEC potential of *Ccl21a*^+^ mTECs possibly suggests additional heterogeneity of bipotent TEC progenitors.

Bipotent TEC progenitors give rise to heterogeneous subpopulations in mTECs. Various mTEC-restricted progenitors have been characterized, leading toward a better understanding of developmental pathways for the generation of diverse mTEC subpopulations. Indeed, reports from single-cell RNA- and protein-profiling analyses predicted various progenitor populations and their transitional intermediates (Baran-Gale et al., 2020; Klein et al., 2023).

mTEC-restricted stem cells, which carry self-renewal properties and long-term mTEC-generating potential, were detected in the Cldn3,4^high^SSEA1^+^ mTEC subpopulation from embryonic and postnatal mice (Hamazaki et al., 2007; Sekai et al., 2014). TECs that express multiple keratin species were also shown to include mTEC-restricted, as well as cTEC-restricted, clonogenic stem cell activity (Bonfanti et al., 2010; Ragazzini et al., 2023). Interestingly, the divergence of mTEC and cTEC lineages in embryonic mouse thymus is generated in the absence of *Foxn1*, suggesting that mTEC-restricted and cTEC-restricted stem cells appear independent of *Foxn1* (Nowell et al., 2011). Recent studies showed that almost all mTECs receive Notch signaling and Cldn3^+^ embryonic mTEC stem cells are absent when Notch signaling is blocked, suggesting that Notch signaling regulates mTEC-lineage specification (Li et al., 2020a; Liu et al., 2020). Another report described that mTEC-restricted progenitors are enriched in the cortico-medullary junction (Onder et al., 2015).

The transcription factor *Relb* is essential for the development of mTECs but is dispensable for cTEC development (Weih et al., 1995; Burkly et al., 1995). *Relb* acts downstream of mTEC-restricted stem cells and is essential for the emergence of RANK^+^ mTEC progenitors, which give rise to AIRE^+^ mTECs (Rossi et al., 2007a; Baik et al., 2016). Most RANK^+^ mTEC progenitors in the embryonic thymus are distinct from CCL21^+^ mTECs, which also show the developmental potential to give rise to AIRE^+^ mTECs (Ohigashi et al., 2024).

A RANK^low^ intermediate stage was noted between mTEC stem cells and RANK^+^ mTEC progenitors (Akiyama et al., 2016). Recently, keratin 19 (*Krt19*)–expressing embryonic TECs were identified as multipotent mTEC progenitors (Lucas et al., 2023). *Krt19*^+^ TECs appear as early as E12.5 before the expression of MHCII, RANK, and CCL21. Fate mapping experiments revealed that *Krt19*^+^ embryonic TECs are capable of long-term generation of mTEC subsets, including *Aire*-expressing mTECs, thymic tuft cells, and CCL21-expression mTECs (Lucas et al., 2023). Like mTEC stem cells, *Krt19*^+^ TECs arise in a *Relb*-independent manner, and a fraction of SSEA1^+^ embryonic TECs express *Krt19*, indicating that *Krt19* expression in embryonic TECs characterizes an initial stage of mTEC-restricted progenitors (Lucas et al., 2023) (Fig. 1 A). Another study reported that mTEC lineage-specific progenitor activity is demonstrated in *Sox9-*expressing embryonic TECs and that high levels of *Krt19* expression are detected in *Sox9*^+^ TECs, suggesting the similarity between *Sox9*^+^ TECs and *Krt19*^+^ TECs as an initial mTEC progenitor (Farley et al., 2023). It is still not clearly understood how a variety of mTEC progenitors are developmentally related and potentially overlapped with each other. For example, it remains unclear whether and how multiple pathways involving distinct progenitors exist in parallel and/or sequentially for the development of AIRE^+^ mTECs, although it seems clear that those progenitors share the developmental potential to become AIRE^+^ mTECs. It will also be interesting to clarify how these progenitors give rise to diverse mTEC subpopulations, including CCL21^+^ mTECs and a variety of mimetic mTECs. In addition, the post-AIRE origin of mimetic mTECs and its Aire dependence are not strict (Michelson et al., 2022), suggesting further diversity in mTEC developmental pathways.

## Thymic epithelial cells and thymus regeneration

Following the generation of cortex and medulla areas, and despite the continued presence of cTEC and mTEC compartments, intrathymic T-cell production throughout the lifecourse is not constant. Indeed, rates of thymus function alter as a result of multiple factors. For example, a gradual decline in thymus tissue during aging reduces thymic size and the intrathymic production and output of naive αβT cells (Palmer, 2013; Venables et al., 2019; Srinivasan et al., 2021). In addition to chronic changes, the thymus is also highly sensitive to stimuli that cause acute damage. Unlike the loss of thymus tissue in aging, acute thymus damage is rapid. Moreover, acute damage can be reversed by the natural ability of the thymus to re-establish following injury. Perhaps of particular significance is thymus recovery following acute damage caused by clinical interventions, which include preconditioning regimes used in cancer treatment (Velardi et al., 2021). Here, thymus regeneration is necessary to rescue patients from T-lymphopenia and prevent life-threatening secondary immunodeficiency. Given the importance of understanding acute thymus recovery for improved immune reconstitution in a clinical setting, our focus here is on the control of thymus regeneration following acute damage for restoration of thymus-dependent T-cell production. We also signpost recent reviews on age-related thymus involution and attempts to regenerate thymus function in the context of aging (Cepeda and Griffith, 2018; de Boer et al., 2023; Li and Zúñiga-Pflücker, 2023; Palmer, 2013).

### Cellular and molecular mediators of thymus regeneration

Most knowledge on mechanisms of thymus recovery comes from experiments using mouse models. Here, injury is typically triggered by either sublethal irradiation (SLI, where thymopoiesis is restored from endogenous lymphoid progenitors) or lethal irradiation followed by hemopoietic stem cell transplantation (HSCT), where thymopoiesis recovers from donor lymphoid progenitors. Both approaches represent robust models where thymus regeneration occurs in response to a temporally controlled injury and have provided insight into regeneration mechanisms.

#### The IL22-IL23 axis

Direct insight into the molecular regulation of thymus repair came from studies of Dudakov and van den Brink (Dudakov et al., 2012), who identified the importance of IL22, a monomeric cytokine belonging to the IL10 family. Following SLI treatment of *Il22*^*−/−*^ mice or transplant of WT bone marrow into lethally irradiated *Il22*^*−/−*^ hosts, they reported diminished cTECs and mTECs as well as reduced CD4^+^CD8^+^ thymocytes, the latter an indicative measure of thymopoiesis (Dudakov et al., 2012). In addition, recombinant IL22 administration enhanced thymus regeneration following damage in WT mice (Dudakov et al., 2012), while increased IL22 levels were observed after damage (Dudakov et al., 2012; Pan et al., 2014). Thus, thymus regeneration is IL22 dependent, and damage provokes increased availability of this key cytokine, the latter also being reported in the context of HSCT in man (Shang et al., 2021). Subsequent studies also demonstrated the importance of IL22 for thymus function in models of Graft versus Host Disease (Dudakov et al., 2017; Pan et al., 2019a). Mechanistically, events upstream of the IL22 requirement mapped to loss of CD4^+^CD8^+^ thymocytes, stimulating IL23 production from DCs, which then triggered IL22 production from Rorγ(t)-dependent ILC3 (Dudakov et al., 2012). Interestingly, in the context of allogeneic HSCT, donor T cells were also reported as an IL22 source (Pan et al., 2019b). Analysis of downstream events indicated IL22 acted directly on thymic stroma by augmenting *Foxn1* expression (Pan et al., 2014), regulating the JAK/STAT/Mcl-1 pathway (Pan et al., 2019a) and promoting TEC survival and expansion (Dudakov et al., 2012). Such observations are important as identification of ILC3 as an important controller of thymus recovery from injury highlights the importance of innate immune components in restoring functionality in an organ important in adaptive immunity. Finally, they also fit well with the ability of IL22 to promote regeneration in multiple organs (Lindemans et al., 2015; McGee et al., 2013; Ren et al., 2010), pointing toward common mechanisms of tissue repair in lymphoid and non-lymphoid tissues.

#### Importance of the TNF receptor superfamily (TNFRSF)

Multiple TNFRSF members regulate steady-state development and function of immune organs. Three receptors in particular, LTβR, RANK, and CD40, regulate TEC development, most notably the mTEC lineage. For example, RANK expression is confined to the mTEC lineage (Hikosaka et al., 2008; McCarthy et al., 2015) and is an essential regulator of AIRE^+^ mTEC development in the embryo (Rossi et al., 2007a), a role it shares with CD40 during postnatal stages (Akiyama et al., 2008; Irla et al., 2008). LTβR, which is expressed by both cTECs and mTECs (Cosway et al., 2017; Hikosaka et al., 2008), influences mTEC cellularity and homeostasis (Boehm et al., 2003; Cosway et al., 2017), including CCL21^+^ mTEC^low^ (Lkhagvasuren et al., 2013) and FEZF2^+^ mTEC (Takaba et al., 2015). Given their importance in homeostatic control of TEC, several studies have investigated roles of LTβR and RANK in thymus regeneration.

Following thymus injury, RANKL increases in expression (Dudakov et al., 2012; Li et al., 2020b; Lopes et al., 2017), including on lymphoid tissue inducer (LTi)/ILC3 cells after lethal total body irradiation (Dudakov et al., 2012; Lopes et al., 2017) and also on both LTi/ILC3 and CD4^+^ thymocytes after SLI (Lopes et al., 2017). Interestingly, augmentation of RANKL expression is transient, with surface RANKL on LTi/ILC3 and CD4^+^ thymocytes returning to baseline by 20 days after damage (Lopes et al., 2017). This suggests a feedback loop occurs during progression toward thymus recovery that negatively regulates RANKL availability. Such a mechanism may be important, as prolonged RANKL availability disrupts TEC microenvironments by skewing toward the mTEC lineage (Hikosaka et al., 2008; Yin et al., 2017). Importantly, provision of RANKL, which expands mTEC in steady-state thymus (Ohigashi et al., 2011), was shown to augment thymus regeneration, while RANKL blockade impaired regeneration (Lopes et al., 2017). Furthermore, administration of ruxolitinib, a Janus kinase (Jak) inhibitor that blocks the Jak-STAT pathway implicated in TEC survival (Lomada et al., 2016), decreased RANK expression and impaired thymus regeneration following SLI (Li et al., 2020b). Collectively, these findings provide strong evidence for a RANK-RANKL axis in thymus repair that involves provision of RANKL by both innate (LTi/ILC3) and adaptive (CD4^+^) immune cells. Interestingly, additional hemopoietic cells, notably γδT cells (Roberts et al., 2012), CD1d-restricted NKT cells (White et al., 2014), and progenitor T cells (Singh et al., 2020), have also been shown to express RANKL, with the latter shown to be effective in regeneration of the aged thymus. It will be interesting to address whether these additional sources of RANKL are important in the restoration of thymus function following acute injury.

Of particular interest are the effects of RANKL administration on thymus regeneration. Here, increases in both cTECs and mTECs were noted in the context of both SLI and HSCT (Lopes et al., 2017). As RANK is expressed by mTECs but not cTECs (Hikosaka et al., 2008) and RANK^+^ TEC progenitors are committed to the mTEC lineage (Akiyama et al., 2016; Baik et al., 2016), this raises the question of how can RANK stimulation aid in cTEC regeneration? One possibility is that in addition to direct effects on the RANK^+^ mTEC lineage, RANKL may trigger the regeneration of RANK^−^ cTEC by stimulating RANK on non-TEC cells. Several lines of evidence are consistent with this. First, RANK is expressed by thymic ILC3/LTi, where it is upregulated following thymus damage (Lopes et al., 2017). Second, RANK stimulation of LTi/ILC3 upregulates their expression of LTα, which is important for the positive effects of RANKL on thymus regeneration (Lopes et al., 2017). Third, LTβR expression by cTECs increases following thymus damage (Lopes et al., 2017). Whether LTα-LTβR interactions between LTi/ILC3 and cTEC and/or cTEC progenitors explain the ability of RANKL to stimulate cTEC recovery requires further examination. Finally, there is further evidence for the importance of LTβR in the recovery of thymus function following damage, with diminished progenitor recruitment to the thymus of *Ltbr*^*−/−*^ mice after SLI (Shi et al., 2016), and LTβR stimulation using agonistic antibodies enhancing thymopoiesis after HSCT (Lucas et al., 2016). Whether these effects map to stimulation of LTβR on TECs or on other non-TEC stromal cells such as mesenchyme and endothelium that also express LTβR is not fully clear. Collectively, these observations indicate the importance of LTβR and RANK in the recovery of thymus function following damage and suggest their requirement involves mechanisms involving some interplay between the two receptors. That both LTβR and RANK are key TEC regulators in the steady state further suggests common mechanisms operate to control both thymus development and regeneration.

#### Type 2 immune mediators

In addition to thymus, several organs can regenerate and regain effective functionality following injury, and it is likely that study of these tissues has aided in elucidating mechanisms of thymus regeneration. Of particular relevance here are components of type 2 immunity, a form of immune response that normally occurs during allergic reactions and following helminth infection (Maizels and Gause, 2023; Molofsky and Locksley, 2023). Type 2 immunity is typically characterized by production of the cytokines IL4, IL5, IL9, and IL13, and involvement of multiple innate cells (e.g., ILC2, mast cells, basophils, and eosinophils). Regarding the influence of these immune mediators on tissue microenvironments, expression of the type 2 IL4 receptor plays an important role. Consisting of IL4Rα, IL13Rα1, and IL13Rα2 components, expression is detectable in epithelial, endothelial, and mesenchymal cells, where it operates as a receptor for both IL4 and IL3 (Gieseck et al., 2018; Junttila, 2018).

Several lines of evidence demonstrate the importance of type 2 immunity in tissue regeneration (Gieseck et al., 2018; Gurtner et al., 2023). For example, in eosinophil-deficient ΔdblGATA mice, where eosinophil development is abrogated as a result of deletion of a high-affinity GATA binding site in the GATA1 promotor (Yu et al., 2002), liver regeneration following either partial hepatectomy or carbon tetrachloride-induced injury is defective. Importantly, this requirement for eosinophils maps at least in part to their production of IL4 (Goh et al., 2013). Interestingly, IL4 targets fibro-adipocyte progenitors that express the type 2 IL4R, a process that inhibits their differentiation toward adipocytes and demonstrates the importance this stromal-expressed receptor in tissue homeostasis and regeneration (Heredia et al., 2013; Lee et al., 2015a). Similarly, eosinophils are recruited following nerve injury and help mediate axon regeneration through their production of IL4 (Liebendorfer et al., 2023; Pan et al., 2020, 2022). Relevant to these observations in other tissues, the adult thymus contains multiple hemopoietic components of type 2 immunity at steady state, including IL4/IL13-producing iNKT cells (Lee et al., 2015b; Miller et al., 2018), IL5-producing ILC2 (Jones et al., 2018; Nussbaum et al., 2013), eosinophils (Albinsson et al., 2023; Cosway et al., 2022; Gatti et al., 2023; Guerri et al., 2013), and basophils (Kim et al., 2010). Also present are stromal cells expressing the type 2 IL4R (White et al., 2017) and stromal subsets producing alarmins capable of triggering type 2 immunity, including IL25^+^ thymic tuft cells (Bornstein et al., 2018; Miller et al., 2018) and IL33 (Ferreira et al., 2021; Xu et al., 2022). This availability of multiple cellular and molecular components of type 2 immunity at steady state raises the possibility of their involvement in regulation of thymus following injury. In line with this, thymic macrophages, eosinophils, and neutrophils are increased following low-dose irradiation that promotes apoptosis induction in CD4^+^CD8^+^ thymocytes (Kim et al., 2010). Moreover, experiments in *Csf1*^*op/op*^, ΔdblGATA, and *Cxcr2*^−/−^ mice indicated a role for thymic innate cells in the clearance of apoptotic cells caused by tissue injury (Kim et al., 2010). However, whether such events were then also connected to the recovery of TEC microenvironments following damage was not assessed.

**Figure 2.**
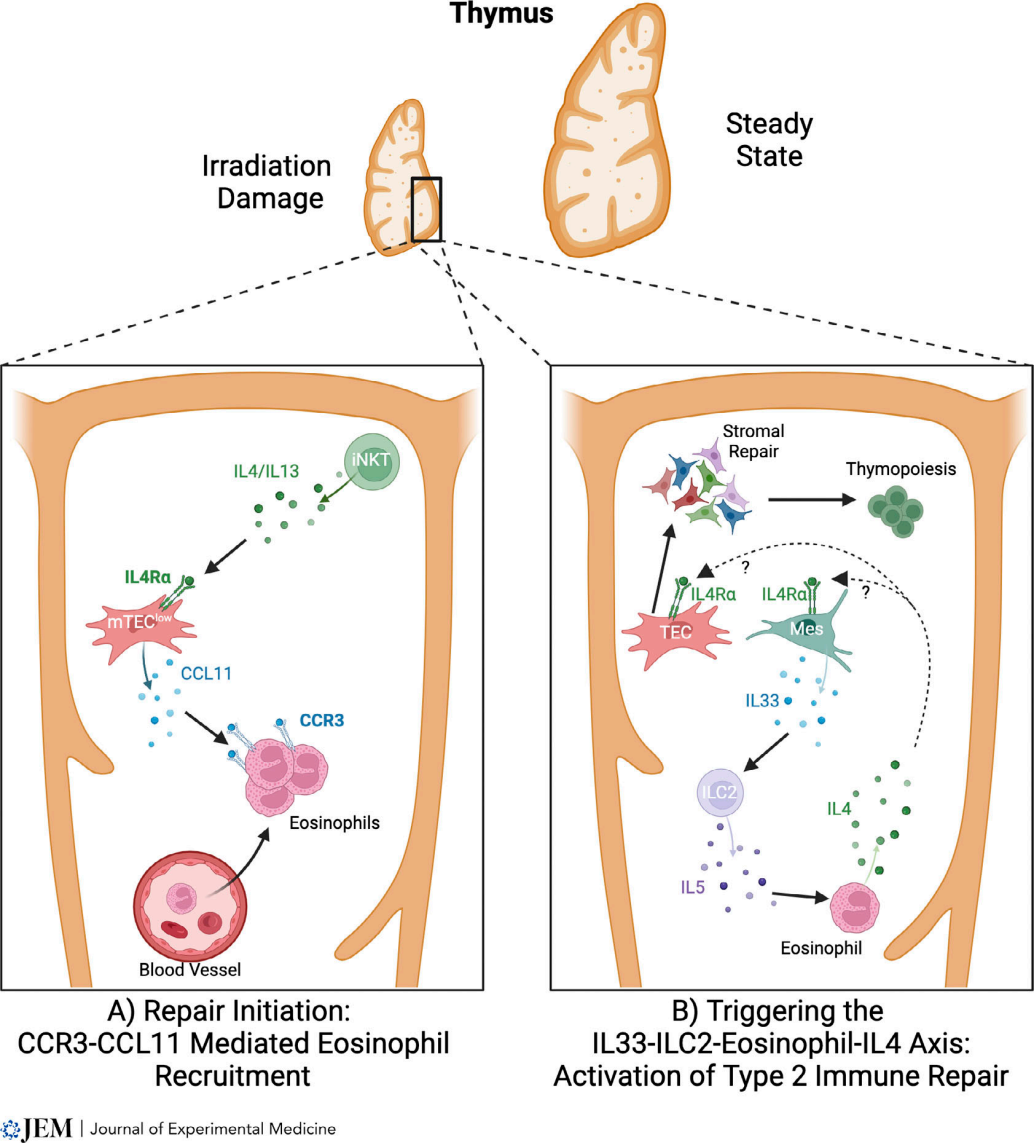
**A type 2 cytokine axis controls thymus regeneration. (A and B)** In models of thymus damage caused by sublethal irradiation, intrathymic iNKT cells are relatively radioresistant compared to their conventional thymocyte counterparts. This enables iNKT-cell secretion of type 2 cytokines to operate on thymic stroma, where it enhances production of the chemokine CCL11 by mTEC. This in turn causes a rapid surge in the recruitment of CCR3^+^ eosinophils from the periphery into the thymus (A). In subsequent stages of regeneration, the alarmin IL33 controls ILC2 that produces IL5, a cytokine important in eosinophil regulation. ILC2 feeds into the requirement for eosinophils in thymus regeneration, where eosinophil production of IL4 triggers recovery of the intrathymic microenvironments that are important for the restoration of T-cell development (B). Although IL4 production by eosinophils is important for thymus generation, whether this cytokine causes TEC generation directly, or indirectly through the targeting of non-TEC stroma such as mesenchyme (Mes), is not clear.

Direct evidence for eosinophil involvement in this process was reported (Cosway et al., 2022), where thymus regeneration was severely impaired in eosinophil-deficient ΔdblGATA mice that received SLI. Here, defective recovery of both cTEC and mTEC lineages occurred, resulting in reduced thymopoiesis. Mechanistically, eosinophil involvement requires their recruitment to the injured thymus (Cosway et al., 2022), which is controlled by CCR3 and its ligand CCL11. This chemokine is produced by several thymic stromal subsets, and at least in the context of intrathymic expression following irradiation, is triggered in thymic stroma by IL4-producing CD1d-restricted NKT cells (Cosway et al., 2022). Such findings point toward the importance of stromal cell expression of the type 2 IL4R, which agrees with defective thymus regeneration also observed in *Il4ra*^*−/−*^ mice (Cosway et al., 2022). Collectively, they suggest a model in which early stages of thymus regeneration involve type 2 cytokine production by NKT cells, which triggers an increase in CCL11 production by type 2 IL4R^+^ thymic stroma, and results in the recruitment of eosinophils to the damaged thymus (Fig. 2 A). ILC2 were identified as important regulators of eosinophil-mediated thymus regeneration through their production of IL5, and events upstream of ILC2 involve the alarmin IL33, whose expression in thymus predominantly maps to mesenchymal cells (Cosway et al., 2023). Involvement of IL33 and ILC2 in thymus regeneration was subsequently confirmed in other studies (Nevo et al., 2024). The requirement for IL33 as a key regulator of ILC2 during recovery from SLI is selective within alarmins, as IL25 administration and IL25 deficiency had no effects on thymus regeneration (Cosway et al., 2023). This is consistent with normal endogenous thymus regeneration following SLI in *Pou2f3*^*−/−*^ mice that lack tuft cells, the sole intrathymic source of IL25 (Cosway et al., 2022; Nevo et al., 2024). Interestingly, thymic tuft cells were reported to aid in thymus regeneration following dexamethasone-induced thymocyte depletion (Nevo et al., 2024), suggesting differing recovery mechanisms following SLI and dexamethasone-induced thymus damage. On the mode of action of eosinophils, IL4 administration improved defective thymus regeneration in ΔdblGATA mice (Cosway et al., 2023), suggesting production of this type 2 cytokine explains at least in part the role of eosinophils in thymus regeneration. These findings extend our understanding of innate immune components in thymus injury and point toward a network in which IL33 production by thymic mesenchyme triggers the expansion of IL5-producing ILC2, which then act upon IL4-producing eosinophils that regulate recovery of thymic stromal microenvironments (Fig. 2 B).

#### Known unknowns in mechanisms of thymus regeneration

**Figure 3.**
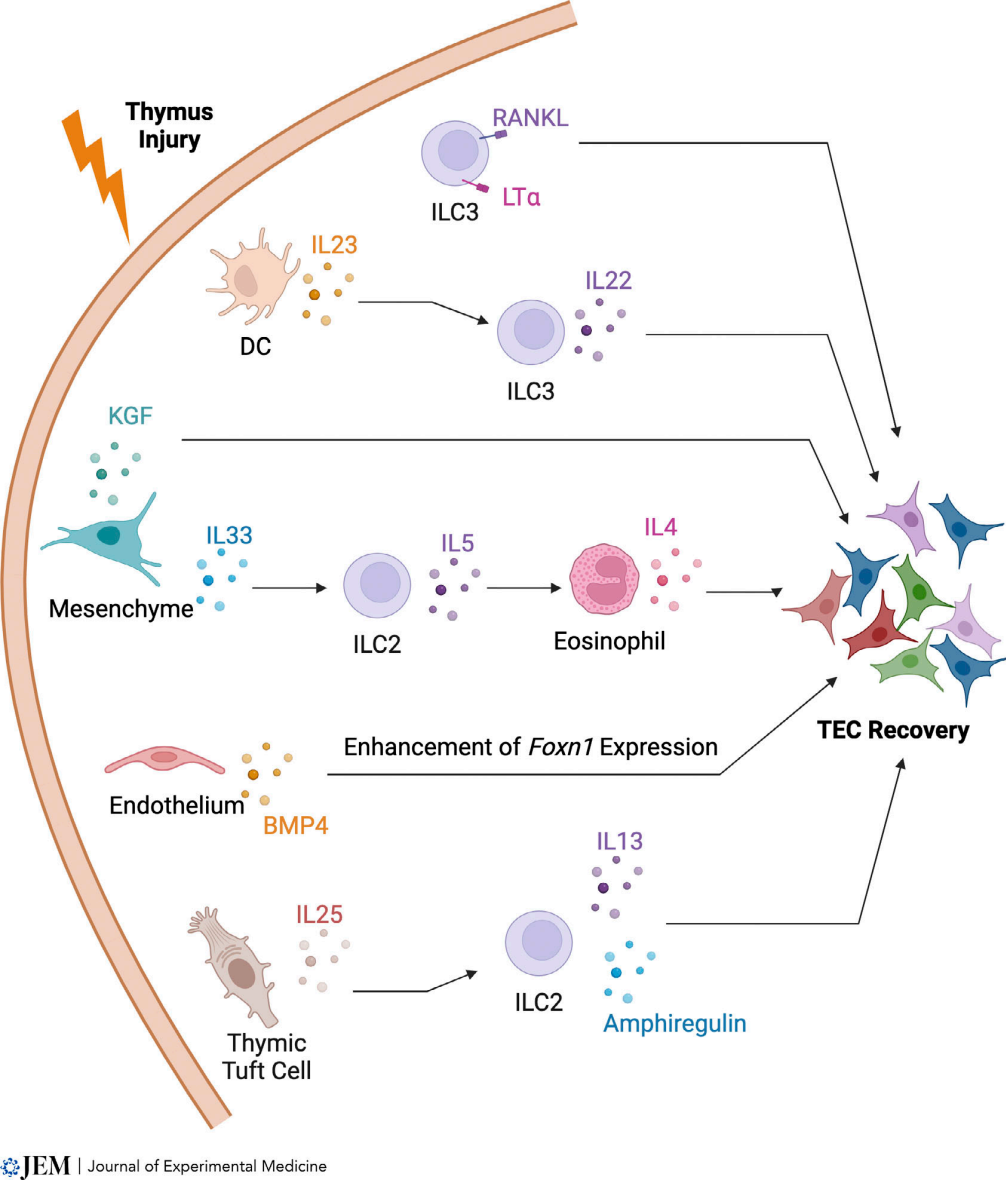
**Multiple pathways control thymus regeneration.** Several pathways have been reported to be important in the recovery of thymus function following damage. It is interesting to note that all pathways shown here contain hemopoietic and thymic stromal elements, in particular cellular components of the innate immune system that include eosinophils, ILC2, and LTi/ILC3. Furthermore, multiple pathways leading to TEC regeneration can share individual cellular elements. However, it remains unclear whether these pathways represent distinct, independent pathways that lead to TEC regeneration, or whether they may intersect and/or operate simultaneously during thymus recovery.

Studies outlined above have provided important insight into the cellular and molecular mechanisms that control thymus regeneration, with a particular focus on the TNFRSF, the IL22 axis, and type 2 immune components (Fig. 3). However, important questions remain regarding the mode of action of these pathways. In particular, while attempts have been made to identify the TEC populations from which cortex and medulla areas re-establish after damage (Dumont-Lagacé et al., 2017, 2020; Ohigashi et al., 2015; Popa et al., 2007; Rode and Boehm, 2012; Rode et al., 2015), the precise TEC target populations of the regeneration mechanisms described here are only partly understood (Cowan et al., 2020). Here, and as performed by Nevo et al. (2024), use of single-cell RNA-sequencing approaches to advance understanding of damage and repair in relation to TEC complexity will be valuable. Indeed, recent studies (Horie et al., 2023) have used this approach to reveal sustained disruption of the mTEC compartment following irradiation-induced damage.

Regarding IL22 involvement, IL22R was shown to be expressed by around 40% of cTEC, mTEC^lo^, and mTEC^hi^ (Dudakov et al., 2012), suggesting not all TEC are IL22 responsive. Moreover, whether the ability of IL22 to increase TEC populations after damage relates to effects on mature cTEC and mTEC and/or TEC progenitors is not clear. It is interesting to note a recent study showing TEC expression of the aryl hydrocarbon receptor (AHR) expression is important in thymus regeneration, where it regulates IL22R expression (Shen et al., 2023). Further definition of IL22R expression on the TEC progenitor populations described above, and the ability of AHR to modulate its expression in TEC subsets, will help to address this.

In relation to the known importance of type 2 immune networks, it is currently not known how IL4 regulates thymus regeneration. Given the broad expression of the type 2 IL4R by thymic stroma, several explanations are possible. For example, IL4 could act directly on TEC/TEC progenitor cells and mediate their expansion, as has been suggested in the context of epithelial regeneration in liver (Gieseck et al., 2016; Goh et al., 2013). Alternatively, IL4 might directly act upon mesenchymal cells which, as known producers of TEC regulators (e.g., fibroblast growth factor and epidermal growth factor [EGF]) (Jenkinson et al., 2003; Shinohara and Honjo, 1996) may then control the re-establishment of TEC populations indirectly. Evidence for such an indirect mechanism of TEC regeneration involving additional non-TEC stromal cells is evident in other reports, including where TEC regeneration is controlled by endothelial cell production of BMP4 (Wertheimer et al., 2018), and expression of MafB by mesenchyme (Hashimoto et al., 2021). Such a mechanism in thymus might also parallel mechanisms seen in other tissues including the intestine, where regeneration via intestinal epithelial stem cells requires EGF production by Paneth cells (Calafiore et al., 2023). Irrespective of whether IL4 acts directly or indirectly on TEC during thymus regeneration, as with the IL22 axis the nature of the TEC/TEC progenitors that may be targets of IL4 is not known. Finally, it is interesting to note both tissue repair (e.g., thymus, gut, liver) and pathological fibrosis share involvement of type 2 immune components (Gieseck et al., 2018). This points toward the need to understand how type 2 immunity is balanced and regulated in the context of tissue repair and damage. They may also highlight potential difficulties in attempts to therapeutically harness components of type 2 immunity for thymus regeneration without provoking unwanted fibrosis pathology. In addition to the pathways of endogenous regeneration discussed in detail here, it is important to emphasize that additional studies focusing on immune reconstitution following HSCT have also been valuable in the identification of mechanisms that govern thymus recovery. For example, both keratinocyte growth factor (Rossi et al., 2007b; Kelly et al., 2008) and p53 inhibition (Kelly et al., 2010; Rodrigues et al., 2017) have been shown to have positive effects TEC survival, proliferation, and function, providing further insight into pathways that control the recovery of thymus function.

A further point to consider in thymus regeneration is to what extent the repaired thymus reacquires all features and functional properties seen in the unmanipulated thymus. While studies typically examine cTEC/mTEC^lo^/mTEC^hi^, it is unclear whether full TEC heterogeneity is restored after damage. For example, examination of the regeneration of mimetic mTEC subsets that play important roles in thymus function including tolerance induction may be important (Givony et al., 2023; Michelson et al., 2022). Relevant to the re-establishment of tolerance, early stages of thymus regeneration after SLI demonstrate an imbalance in Sirpα^+^ and Sirpα^−^ DC subsets (Michaels Lopez et al., 2022). At least in the context of HSCT, mTEC deficiencies also occur, which cause a breakdown in central tolerance mechanisms (Alawam et al., 2022). Uncertainties also surround the ability of the regenerated thymus to support production of qualitatively distinct T-cell lineages. While most studies focus on recommencement of conventional CD4^+^ and CD8^+^ αβT-cell development, whether thymus regeneration results in the effective production of non-conventional T cells, including NKT cells, Eomesodermin^+^ memory phenotype CD8^+^ T cells, CD8αα intra epithelial lymphocytes, mucosal associated invariant T cells (Ashby and Hogquist, 2020; Pellicci et al., 2020), and γδ T cells, is less well studied.

## Concluding remarks

Advances in understanding TEC development and repair provide important insight into multiple aspects of thymus biology. There is agreement that cTEC/mTEC generation and maintenance are controlled by TEC progenitors that are present in fetal life and persist into adulthood. In addition, repair of the injured thymus to restore T-cell production requires multiple cellular and molecular components of the innate immune system. These latter findings draw interesting parallels with similar repair mechanisms in other non-lymphoid tissues and organs.

Despite this progress, a full understanding of TEC development and the precursor-product stages it contains is lacking. This is perhaps explained at least in part by the remarkable increase in TEC heterogeneity revealed by single-cell RNA-sequencing studies. Here, it is important to note that developmental properties and functional importance of some of these newly identified TEC subsets have yet to be analyzed. Experiments examining precursor-product relationships of defined TEC subsets, perhaps through use of either fate mapping or in vitro reaggregation cultures, should help redraw clearer TEC developmental pathways. A better understanding of TEC heterogeneity will also help understand how TEC microenvironments recover following injury. While regulators of thymus regeneration are known to include innate immune components, the TEC populations that are targets of innate immune control remain poorly understood. Understanding the influence of the innate immune system on progenitor TECs and/or their mature progeny may aid targeted approaches to therapeutically control thymus function in a clinical context.
