# Correction: Generation and repair of thymic epithelial cells

**DOI:** 10.1084/jem.2023089409042024c

**Published:** 2024-09-12

**Authors:** Graham Anderson, Emilie J. Cosway, Kieran D. James, Izumi Ohigashi, Yousuke Takahama

Vol. 221, No. 10 | https://doi.org/10.1084/jem.20230894 | July 9, 2024

The authors regret that the original description at the bottom of Fig. 2 A contained a typographical error. The corrected figure is shown here with the caption “CCR3-**CCL11** Mediated Eosinophil Recruitment.” This correction does not change the original conclusions of the review, and the figure legend remains unchanged. The error appears in PDFs downloaded before August 28, 2024.

**Figure 2.**
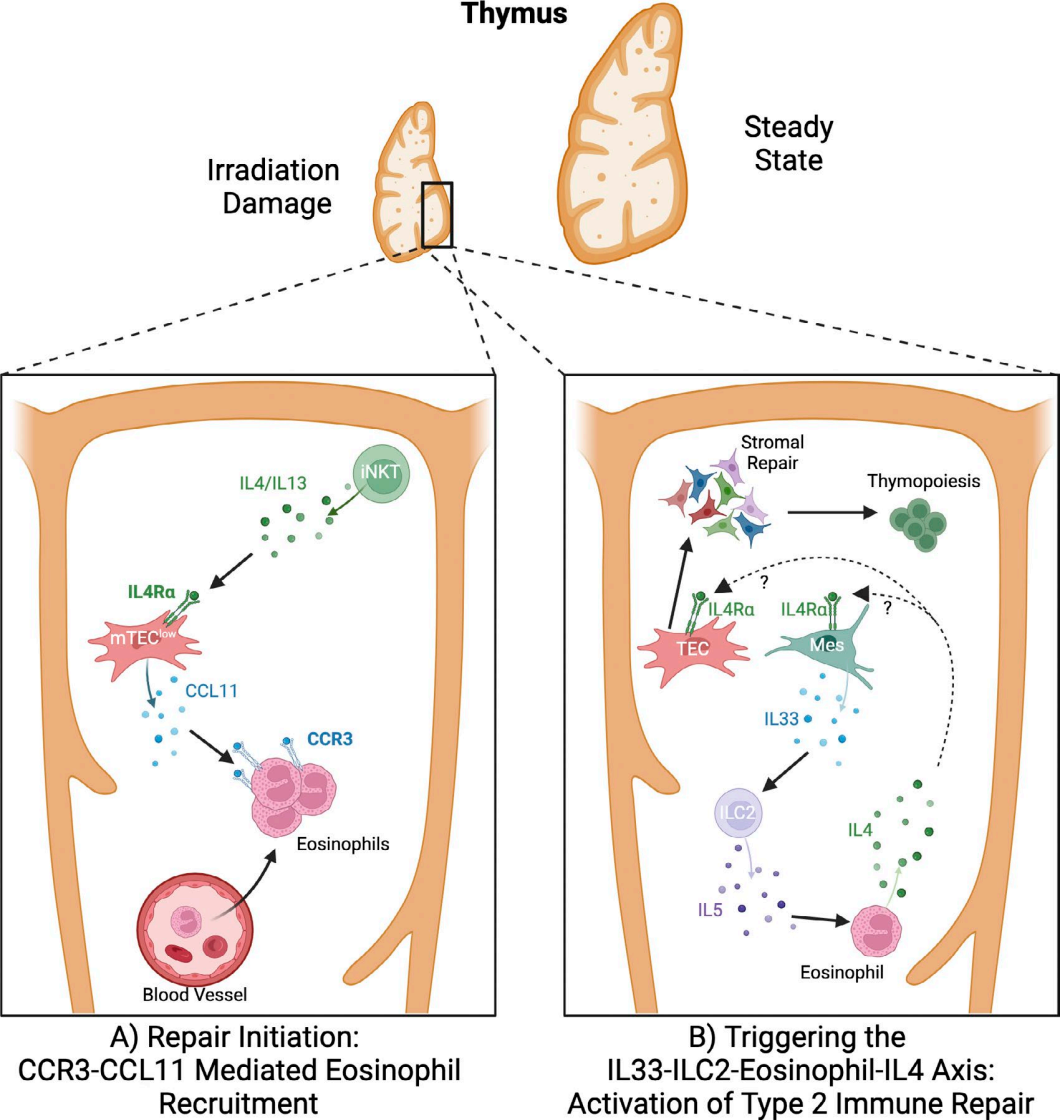
A type 2 cytokine axis controls thymus regeneration.

